# Using brain stimulation to disentangle neural correlates of conscious vision

**DOI:** 10.3389/fpsyg.2014.01019

**Published:** 2014-09-23

**Authors:** Tom A. de Graaf, Alexander T. Sack

**Affiliations:** ^1^Department of Cognitive Neuroscience, Faculty of Psychology and Neuroscience, Maastricht UniversityMaastricht, Netherlands; ^2^Maastricht Brain Imaging CentreMaastricht, Netherlands

**Keywords:** NIBS, TMS, TES, tDCS, visual awareness, consciousness, NCC

## Abstract

Research into the neural correlates of consciousness (NCCs) has blossomed, due to the advent of new and increasingly sophisticated brain research tools. Neuroimaging has uncovered a variety of brain processes that relate to conscious perception, obtained in a range of experimental paradigms. But methods such as functional magnetic resonance imaging or electroencephalography do not always afford inference on the functional role these brain processes play in conscious vision. Such empirical NCCs could reflect neural prerequisites, neural consequences, or neural substrates of a conscious experience. Here, we take a closer look at the use of non-invasive brain stimulation (NIBS) techniques in this context. We discuss and review how NIBS methodology can enlighten our understanding of brain mechanisms underlying conscious vision by disentangling the empirical NCCs.

## INTRODUCTION

The search for neural correlates of consciousness (NCCs) continues. While “consciousness” has been a philosophical and scientific topic of interest throughout the ages, the surging development of brain research technology has caused something of a renaissance in the last quarter century. Quick advancements in functional magnetic resonance imaging (fMRI) and electro-/magnetoencephalography (EEG/MEG), combined with seminal contributions from high-profile pioneers (e.g., Crick and Koch, 1990), provided the NCC research program in humans with quite some momentum (Dehaene and Naccache, 2001; Rees et al., 2002a; Crick and Koch, 2003; Koch, 2004), while groundbreaking animal experiments were performed as well (Cowey and Stoerig, 1995; Leopold and Logothetis, 1996). Today, the neuronal mechanisms underlying “visual awareness,” “conscious perception,” or “subjective experiences,” are regular topics in empirical research.

## EMPIRICAL NCCs

### CONSCIOUSNESS

No article on “consciousness” is likely to be very meaningful without a clear delineation of what mental faculties exactly are referred to. We previously outlined our preferred rough taxonomy of types of consciousness (de Graaf et al., 2012b). It included (1) self-awareness, (2) higher-order consciousness, (3) “medical awareness” or state-consciousness, and (4) “consciousness-as-experience” or content-consciousness.

*Self-awareness* is the overarching concept of a continuous and controlling self, a being that is defined by the contrast to surroundings and other beings. Research clustered under self-awareness could include such topics as self-recognition, agency, and awareness and situation of the persona inside the body.

*Higher-order consciousness* in our schema is the somewhat folk psychological conception of consciousness, where the abilities to think, reason, and reflect are crucial. It involves typically human faculties such as the realization of past and future, the ability to “think about thinking,” and is likely analogous to, for example, reflective consciousness (e.g., Edelman and Tononi, 2001).

*Medical awareness*, which for simplification we may also refer to as the more common “state consciousness,” is a conception of consciousness as a certain state of being. Patients in a coma or under anesthesia may not be in a conscious state, people under the influence of drugs may be in an alternative conscious state. It is required to be in at least some minimal state of consciousness to achieve:

*Consciousness as experience,* which for simplification we may refer to as the more common “content consciousness.” At any moment in time, provided we are awake (or at least dreaming), we have experiences. They include phenomenal properties (the “what-it-is-like” of the experience) and psychological or “access” properties (the abilities to report, remember, or act on the experienced information; Chalmers, 1996; Block, 2005).

### NCC PARADIGMS

**FIGURE 1:**
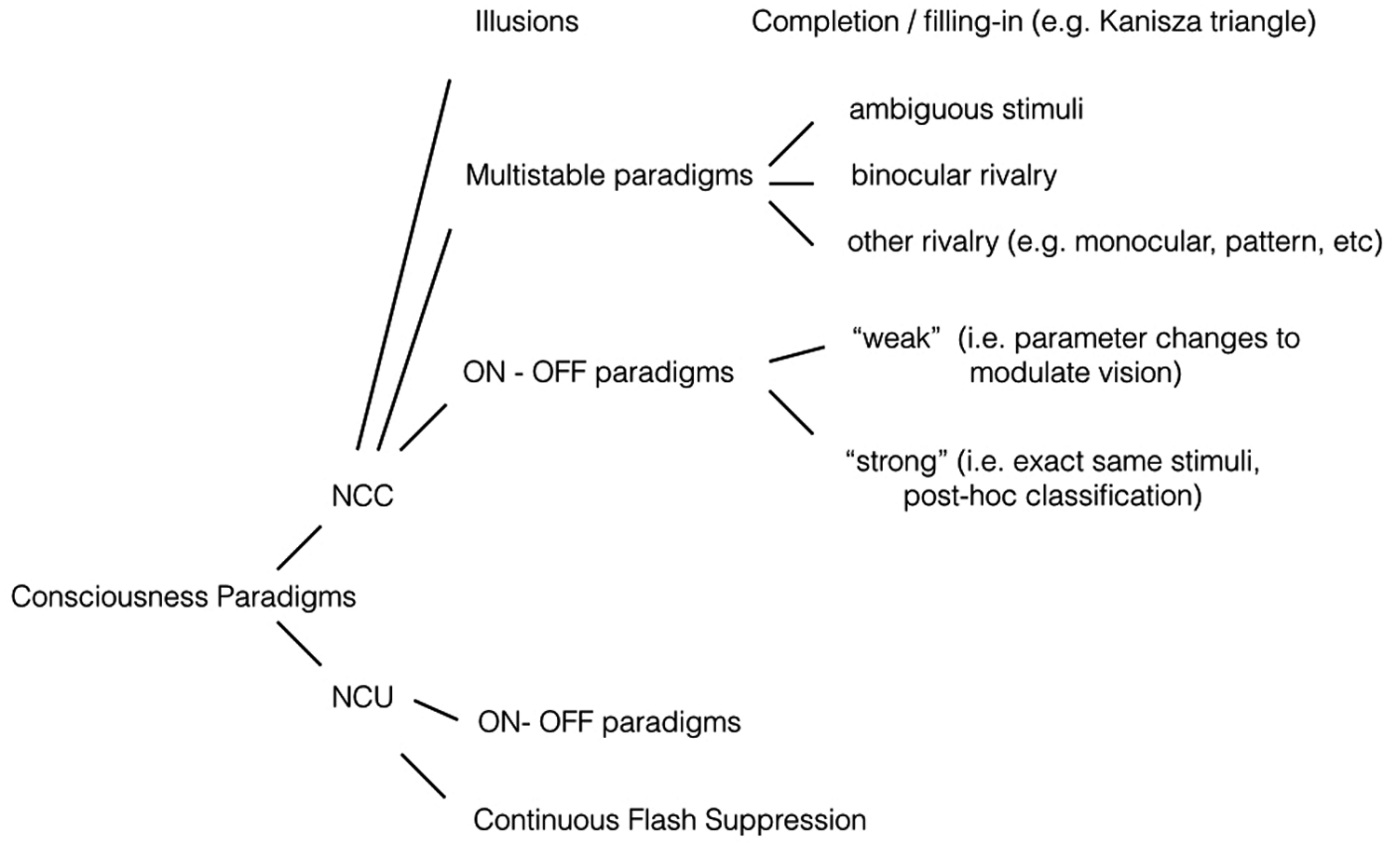
**Experimental paradigms.** NCC (neural correlates of consciousness) paradigms. *Illusions*: If an illusion is defined as a conscious percept that is created endogenously, as opposed to exogenously, then it may serve as a useful NCC stimulus. In most cases, small parameter changes will extinguish the illusion. With for example the Kanizsa triangle, the conscious percept of a triangular outline disappears if the corner elements are rotated. The presence or absence of the conscious percept can be correlated to brain activity. Another form of illusion is filling-in, in which a constant peripheral stimulus is sometimes perceived and other times not. *Multistable paradigms*: Constant visual stimulation leads to a changing conscious percept. A well-known example is binocular rivalry, in which both eyes receive incompatible images and conscious perception fluctuates between the two stimuli. Some brain processes will covary with perception, others will not. *ON–OFF paradigms*: Paradigms in which sometimes a stimulus is perceived, and other times not. It thus involves presence vs absence of a conscious percept, as opposed to presence of percept A vs presence of percept B as in multistable paradigms. The strong version involves no changes in stimulation, the weak version does involve parameter changes. For more details see main text. *NCU (neural correlates of “unconsciousness”) paradigms*. *ON–OFF paradigms*: An ON*–*OFF paradigm can be used for NCC studies if brain activity is contrasted in the ON vs the OFF condition. The same stimuli and setup can be useful for NCU studies, if brain activity is contrasted in the OFF condition vs rest. In other words; which brain processes still obtain if a stimulus is presented but not consciously perceived? *Continuous flash suppression*: A variant of the binocular rivalry paradigm, in which conscious perception is heavily biased towards one eye through repeated salient stimulation of that eye, while the second eye receives a weaker stimulus. That weaker stimulus of interest is thus suppressed for prolonged periods of time, allowing analysis of brain processes nevertheless responding to it.

Early on, pioneering consciousness researcher Baars (1989) pointed out that NCCs can be obtained by contrasting conditions with conscious experience to conditions without conscious experience (the *contrastive method*, e.g., Aru et al., 2012). Using neuroimaging paradigms, such as fMRI or EEG, one can contrast these two experimental conditions to isolate the brain mechanisms specific to the conscious condition. Ideally the conscious condition and the non-conscious condition differ from each other as little as possible in terms of stimulation parameters. Over the years, a range of paradigms has been developed for NCC research that allows useful contrasts with no, or minimal, changes in stimulus parameters. Outlined in **Figure 1**, we have developed informally our taxonomy of NCC research paradigms (also presented in de Graaf and Sack, in press). We make no claim to either exhaustiveness or originality/priority in this regard (see e.g., Kim and Blake, 2005), it is simply a grouping that we have found useful to maintain an overview of the many paradigms in NCC research.

One main division is between paradigms to obtain NCCs and paradigms that research neural correlates of unconsciousness (NCUs). The latter are not always referred to as such, but investigations of brain activity elicited by inputs that do not make it to consciousness are clearly valuable in the greater scheme of NCC research. For example, patients with (often right) parietal damage may fail to consciously see (report) stimuli in the opposite visual field (“neglect”), especially when bilateral stimuli are presented (“extinction”). Yet brain imaging studies uncovered activity in early and extrastriate visual regions, in response to these unseen stimuli (Rees et al., 2000, 2002b; Vuilleumier et al., 2001). Goebel et al. (2001) studied fMRI activation in extrastriate regions in blindsight patients. Blindsight is another neuropsychological condition with relevance to consciousness, since in this condition with damaged (connections to) primary visual cortex, patients can correctly report on (“guess”) various features of stimuli that are not consciously perceived (Weiskrantz, 2009). One patient studied by Goebel et al. (2001) experienced conscious motion perception in only one visual hemifield, even though bilateral hMT/V5 cortices were intact. Interestingly, activity in hMT/V5 in both hemispheres was nearly identical with respect to BOLD responses to contralateral visual stimulation. In other words, sustained hMT/V5 BOLD activity did not seem to reflect the presence or absence of visual awareness.

Neural correlates of unconscious processing can also be studied in fully intact brains. In one clever fMRI experiment house and face stimuli were presented either consciously (congruent dichoptic stimulation: e.g., a green house on red background presented to both eyes) or not consciously (incongruent dichoptic stimulation: e.g., a green house on red background in one eye and a red house on green background in the other eye “canceled each other out” at the binocular level). “Face areas” and “place areas” of the brain still responded (although to a lesser extent) to completely invisible pictures (Moutoussis and Zeki, 2002). As a second example, an influential study (Dehaene et al., 2001) could show that even words are processed in extrastriate regions when they are not consciously perceived due to visual masking. In some extrastriate regions, the effects were moreover case-independent. Collectively, these experiments seem to demonstrate that activation in specialized higher-order visual regions is not in itself sufficient for conscious perception. Today, an increasingly popular paradigm that can be used to study NCUs is continuous flash suppression (CFS; Tsuchiya and Koch, 2005). This useful variation to the classical binocular paradigm, in which a stimulus in one eye is suppressed by salient flashing patterns of stimulation in the other eye, can be implemented to suppress visual stimuli for very long durations.

A second main distinction that may be useful is between “strong” and “weak” paradigms. “Strong” paradigms in this context allow changes in conscious percept, or variations in conscious percept, without *any* changes in stimulation parameters. “Weak” paradigms, in contrast, implement small changes in stimulus parameters to determine conscious content. This difference is easily understood in the context of “ON–OFF paradigms,” where stimuli are consciously perceived (ON) or not (OFF). For example in visual masking, experimenters can determine, through the timing between targets and masks, whether targets will be perceived or not (Breitmeyer and Ogmen, 2006). Since this involves a change in stimulus parameters, it is a “weak” ON–OFF paradigm. Alternatively, in a “strong” ON–OFF implementation the stimulus parameters could be fixed at some threshold level, relying on spontaneous neuropsychological fluctuations to lead to a conscious percept on some trials (ON) but not on other trials (OFF).

“Strong” and “weak” refer only to the level of isolation of brain processes in relation to visual awareness, not the scientific value of the paradigm. While constant stimulus parameters allow a “cleaner” isolation of the brain processes related to conscious perception, weak paradigms afford the experimenter control and certainty about the presence, absence, or contents of visual awareness. Both thus have their advantages and disadvantages, and appropriate applications depend on experimental question and brain imaging setup.

All of the various paradigms in **Figure 1** could yield a number of brain processes correlated to conscious vision, including BOLD activations, fMRI connectivity patterns, EEG/MEG event-related potentials, changes in oscillatory power or phase coherence, and so on. All such brain mechanisms would, by definition, correlate to conscious contents. And they would therefore, in the literal sense, be NCCs: neural processes that correlate to consciousness. But in the conceptual and philosophical domain, “NCC” can have quite a specific and involved meaning. So from now on, we refer to such experimental findings strictly as *empirical NCCs.*

## CORRELATES AND “TRUE CORRELATES”

After all, another, or perhaps “true,” meaning of NCC’s has traditionally been the *actual* brain mechanisms responsible for conscious perception. Definitions abound, but an influential definition of a neural correlate of consciousness comes from Chalmers (Chalmers, 2000):

An NCC is a minimal neural system N such that there is a mapping from states of N to states of consciousness, where a given state of N is sufficient, under conditions C, for the corresponding state of consciousness. (Chalmers, 2000, p. 31)

Clearly, “NCC” here is much more refined and constrained than the “empirical NCCs” obtained in neuroimaging research using the contrastive method.

This realization has quite a long history, as pointed out by Miller (2007, p. 162). For instance, Crick (1994) noted: “it does not follow that these particular neurons are the real seat of awareness. They may by their firing, influence other neurons... that are the *true* correlates of awareness” (Crick, 1994, p. 218). Logothetis (1998, p. 541) asked: “Do neurons responding only when a stimulus is perceived actually mediate the conscious experience of this stimulus?” He pointed out that, although his data favored such an interpretation, they “cannot prove it unequivocally” (Logothetis, 1998). There have been others (e.g., Revonsuo, 2000) who noted what Miller (2001, 2007) calls the “constitution/correlation problem;” brain processes that correlate to conscious perception may not necessarily be constituent of that conscious experience. Koch, lastly, points out that it makes sense to distinguish “core NCCs” from “total NCCs,” where core NCCs are responsible for the contents of conscious experience, whereas the total NCC reflects the core NCC plus all enabling factors and is thus required as a whole to obtain a particular conscious experience (Koch, 2004, as discussed in Block, 2005).

## THREE ROLES FOR EMPIRICAL NCCs

In fact, increasing numbers of philosophical (Revonsuo, 2001; Noë and Thompson, 2004; Block, 2005; Miller, 2007; Hohwy, 2009; Neisser, 2012) and empirical researchers (Miller, 2001, 2007; Koch, 2004; Bachmann, 2009; Melloni et al., 2011; Aru et al., 2012; de Graaf et al., 2012b; Kanai and Tsuchiya, 2012; Sergent and Naccache, 2012) have been coming to the conclusion that empirical NCCs are only part of the way there. To make this explicit, as a prelude to outlines for future research opportunities, two similar review papers (Aru et al., 2012; de Graaf et al., 2012b) recently focused on the three fundamentally distinct functional roles that any (part of an) empirical NCC resulting from the contrastive method could reflect. They are *neural prerequisites, neural consequences,* or *neural substrates* of conscious experience.

### NEURAL SUBSTRATES

Neural substrates of a particular conscious experience are the brain events that *directly* caused (epiphenomenalism), reflected (dualism), or were identical with (materialism) the phenomenal experience in our experiment. They were both necessary and sufficient. “Sufficient” in the sense of Chalmer’s definition: only these brain events were required for the experience and nothing more was needed. They were “necessary” only in our current empirical situation (imagine any concrete NCC neuroimaging experiment), and in a non-philosophical sense (“necessary” is rather a loaded term in the context of consciousness), because without these brain events the experience would not have occurred. Hypothetically, perhaps other brain events could have served as substrates for the same, or a similar experience. But in our experiment, it was these brain processes that instantiated the experience. In the current context, there is no clear difference between what we have called “neural substrates,” and the “NCC” of Chalmers, the “real” NCC, “true” NCC, or the “NCC-proper.” We refer to it as “neural substrate of conscious experience” to differentiate it from all the other different types of NCCs discussed here, and to remain consistent with our earlier outlines (de Graaf et al., 2012b; de Graaf and Sack, in press).

### NEURAL PREREQUISITES

Neural prerequisites of consciousness are brain events that are necessary for the conscious experience to occur, but not sufficient. They are *empirical* NCCs, since they consistently co-vary with conscious experience. That is because, in the implemented experimental setup, in the real world, the neural substrates do not arise without them. But importantly, if somehow through some hypothetical and counterfactual scenario the neural substrates *did* arise without the prerequisites, the conscious experience would be there and unchanged. To understand this, one might imagine highly advanced brain stimulation techniques targeting specifically and only the neural substrates. Or, somewhat more realistically, a different experimental paradigm could give rise to the same neural substrates of a conscious experience, via a different route and thus through different neural prerequisites. Or perhaps it would be possible to attain an identical conscious experience in a dream, without any external stimuli, leaving out some neural prerequisites that obtained in our experiment. In sum, different chains of brain events might have served as prerequisites to the same neural substrates. But, coming back to our perspective as an empirical researcher, we are talking about *empirical* NCCs that factually *did* result from a concrete brain imaging experiment with a given experimental paradigm. And we are outlining possible functional roles of these empirical NCCs. So, in the empirical situation at hand, some of our empirical NCCs could, and likely would, have functioned as neural prerequisites of the studied conscious experience, rather than being the neural substrates. It is useful to distinguish these functional roles.

Expanding on our previous outline (de Graaf et al., 2012b) and as discussed in detail in de Graaf and Sack (in press), it may be useful to further distinguish two possible “types” of neural prerequisites: *content-invariant* prerequisites, and *content-specific* prerequisites. Content-invariant prerequisites would co-occur with any conscious experience. Much of the findings on brain events enabling a conscious state would pertain to content-invariant prerequisites. For example, connectivity between reticular formation and precuneus might be required for any conscious experience to arise (Silva et al., 2010). Content-invariant prerequisites are interesting, but would be relatively easy to identify by eliciting different contents of consciousness across different NCC paradigms and seeing which empirical NCCs are consistently observed.

Content-specific prerequisites would be much more tricky to dissociate from neural substrates, since they co-occur, by definition, with and only with each occurrence of a particular conscious experience. For example, a particular visual image presented for a sufficiently long duration will consistently lead to a specific conscious experience of it, but also to a (in part) specific cascade of non-conscious feature processing steps in early visual regions. To complicate things further, in reality the distinction between content-specific and content-invariant prerequisites may not be a dichotomy, but something of a continuum. There may be modality-specific prerequisites, feature-specific prerequisites, perhaps even concept-specific prerequisites, and so on.

As a concrete example, some binocular rivalry results have implicated primary visual cortex in conscious vision (Polonsky et al., 2000; Tong and Engel, 2001), and we learn from blindsight patients that this region appears to be crucial for conscious vision (Weiskrantz, 2009). Yet, Crick and Koch (1995) argued that primary visual cortex is unlikely to be part of neural substrates (“true correlates”) of consciousness. Primary visual cortex activation may therefore be a neural prerequisite, and perhaps particular processes within it content-specific prerequisites. As Silvanto (2008) argues, these processes may be crucial for conscious vision to arise, even though the conscious experience is localized elsewhere in the brain.

In another example, Beck et al. (2006) reported on the role of parietal cortex in change blindness. Finding that parietal rhythmic transcranial magnetic stimulation (rTMS) slowed reaction times to change and reduced the proportion of detected changes, they concluded *“It is important to note that we are not arguing that the parietal cortex is the neural locus of consciousness, but rather that the functions associated with parietal cortex, such as attention and visual short term memory (VSTM), may be necessary prerequisites to visual awareness”* (Beck et al., 2006, p. 716). They suggested that the functional relevance of parietal cortex should be tested in other paradigms to determine whether this role is general rather than specific to change blindness (in our terminology; whether it is a content-invariant prerequisite). As a clear prelude to discussing the value of non-invasive brain stimulation (NIBS) in the current framework, we may focus on another quote from the Introduction of the same article. After discussing prior neuroimaging work: *“In all these studies, it remains possible that the parietal activity found was a consequence of subjects’ awareness and did not play either a necessary or causal role in producing that awareness”* (Beck et al., 2006, p. 712).

### NEURAL CONSEQUENCES

There may be brain events that consistently co-occur with a conscious experience, that are neither necessary nor sufficient for the experience to arise. They are not substrates, and they are not even required for the substrates to arise, so they would – with regards to the phenomenal experience – not be missed. Yet, they are there, because they consistently follow a conscious experience.

Again, consequences can be content-invariant or content-specific. Content-invariant consequences would be empirical NCCs across the range of contents of consciousness. They could include attention effects, if the simple occurrence of an experience grabs your attention, or response preparation and memory processes (we’re evolved to act on and learn from consciously perceived information). Consequences can therefore be – while useless to the neural substrates of a conscious experience – rather useful for the organism. Citing Seth (2009), Aru et al. (2012) point out that, indeed, meaningful neural consequences of conscious experience are a logical consequence of assigning any functionality to conscious perception. As with prerequisites, while content-invariant consequences could be isolated through variations in NCC paradigms and stimuli, content-specific consequences are more difficult to distinguish from neural substrates. If a picture of a beach elicits in me strong emotional memories of a long-lost friend, stimuli depicting beaches could consistently elicit in me a cascade of brain events that would not only be content-specific, but even participant-specific.

## DISENTANGLING EMPIRICAL NCCs

There are thus three fundamentally different roles one might assign to (part of) any empirical NCC. And things may become even more complex, if it turns out that combinations of and interactions between content-specific and content-invariant brain events are responsible for conscious experiences. That would make separations of neural prerequisites and substrates difficult, or even somewhat arbitrary. Therefore, as new evidence continues to inform neurobiological models of consciousness, we should follow the advice of properly thinking through the cascade of brain events that underlies conscious experience, reframing the question as we go (Hohwy, 2009; Feinberg, 2012; Neisser, 2012). This way, theoretical and computational models will become increasingly sophisticated (Dehaene et al., 2003; Dehaene and Changeux, 2011; Oizumi et al., 2014). For the moment, however, research with the contrastive method (Aru et al., 2012), using paradigms such as those outlined in **Figure 1**, continues to specify and increasingly constrain empirical NCCs. There are different strategies to try and disentangle the functional roles of empirical NCCs (Aru et al., 2012; de Graaf et al., 2012b). In the remainder of this article, we focus on the contributions of NIBS in this context.

### EMPIRICAL NCCs

Before continuing on to our review of NIBS as a tool for NCC research, it is useful to provide a very quick and rough overview of some empirical NCCs that have been obtained in neuroimaging research. These form, after all, the “starting point” for brain stimulation experiments to probe functional roles of specific empirical NCCs.

While oversimplified, it seems fair to claim that *early visual cortex* (by which we mean V1, V2, V3), certain *extrastriate visual/temporal cortices*, *parietal cortex*, and *frontal cortex* have been linked to conscious vision. We have above already mentioned important studies demonstrating relations between visual awareness and early visual regions. The relevance of increasingly modular extrastriate cortices in vision in general is uncontroversial, and for example bistable vision paradigms have shown that extrastriate regions such as the fusiform face area (FFA) or the parahippocampal place area (PPA) reflect conscious percept rather than the constant visual input (Tong et al., 1998). Bistable paradigms (Kleinschmidt et al., 1998; Lumer et al., 1998; Lumer and Rees, 1999; Sterzer et al., 2002), but also masking (Dehaene et al., 2001), and other NCC paradigms (Rees et al., 2002a; Rees, 2007) have generally reported frontoparietal activations in fMRI. Frontoparietal activations are stronger to trials in which stimuli are consciously seen versus not seen (e.g., Dehaene et al., 2001; Lau and Passingham, 2006), are time-locked to specifically endogenous perceptual switching (Kleinschmidt et al., 1998; Lumer et al., 1998), and are related to attentional/perceptual effects often mentioned in the context of consciousness such as change blindness (Beck et al., 2001) and attentional blink (Marois et al., 2000). The following section will describe how NIBS has contributed to our understanding of the functional roles of these empirical NCCs.

## NON-INVASIVE BRAIN STIMULATION

### BASICS OF NIBS

Non-invasive brain stimulation includes primarily TMS and transcranial electric stimulation (TES), the latter including transcranial direct current stimulation (tDCS) and transcranial alternating current stimulation (tACS).

Transcranial magnetic stimulation involves a strong capacitor linked to a coil. A single TMS pulse with the popular figure-eight coil requires a brief electrical current through the overlapping windings inside the coil, which leads to a short, focal, rapidly changing magnetic field extending perpendicularly from the TMS coil. If the coil is placed tangentially on a human head, this magnetic pulse extends into the brain where it induces an electrical field in neural tissue and ultimately causes action potentials (Wassermann et al., 2008). A single pulse can stimulate neurons at rest, with observable behavioral (motor response; e.g., Barker et al., 1985) or perceptual (phosphenes; e.g., Marg and Rudiak, 1994; Kammer, 1999) consequences. A single pulse administered to a region during processing can disrupt the spatiotemporal organization of regional processing and thus induce behavioral (Pascual-Leone et al., 1991), cognitive (Sack et al., 2002a,b), or perceptual (Amassian et al., 1989) impairments (Pascual-Leone et al., 2000). Multiple TMS pulses in a rhythmic sequence, rTMS, can not only impair function online, but also have effects on cortical excitability outlasting the stimulation protocol (Robertson et al., 2003). More complex and powerful new protocols have been developed [theta-burst stimulation (TBS); Huang et al., 2005], but traditionally simple repetitive stimulation of a cortical region with low frequency (∼1 Hz) has been shown to result in decreased cortical excitability while stimulation at high frequency (∼5 to 20 Hz) has been shown to result in increased cortical excitability (Dayan et al., 2013).

TES can have similar effects on cortical excitability. It involves a power source connected to (minimally) two electrode patches. tDCS refers to a continuous flow of low-intensity electrical current (typically around 1–2 mA) from one electrode patch (anodal) to the other (cathodal). Cathodal stimulation of a brain region hyperpolarizes membranes, ultimately resulting in decreased cortical excitability, while anodal stimulation depolarizes membranes, ultimately resulting in increased cortical excitability (Nitsche and Paulus, 2011; Paulus, 2011). Thus, depending on stimulation parameters both rTMS and tDCS can increase or decrease the excitability, and therefore efficacy of contribution, of any cortical region close enough the surface of the brain (Dayan et al., 2013). That includes a great number of empirical NCCs to target. Another form of TES is tACS. In tACS the electrical current does not flow continuously from one electrode to the other, but instead flows rapidly back and forth between the two, reversing direction at an externally fixed frequency. As we will see below, this can be used to modulate oscillatory brain activity. There are still other implementations of TES (e.g., transcranial random noise stimulation), but these fall outside the scope of the current review. Collectively, TMS and TES can be referred to as NIBS, which has already been, and likely still will be, of great value to NCC research.

### THE NIBS CONTRIBUTION

What is the added value of NIBS, over and above the contrastive method of neuroimaging with the paradigms described above? With neuroimaging, it is difficult to conclude whether or not a region or process is functionally relevant. It remains an open question whether or not, for example in fMRI, a regional BOLD response reflects neural processing that is imperative for the task at hand. For conscious vision, this means it is hard to know whether the conscious percept would have been the same if this particular BOLD response had not obtained. In contrast, manipulating brain activity directly with NIBS as an independent variable, and then evaluating the effects on conscious vision, *does* allow one to draw such conclusions on functional relevance. If a conscious percept is abolished, because a brain region is disrupted, then one way or another the disrupted region was functionally relevant to conscious vision. We do think that advanced neuroimaging paradigms and analyses (e.g., connectivity analysis; Friston et al., 2003; Roebroeck et al., 2005; Friston, 2011) can actually make substantial contributions to separating neural substrates, prerequisites, and consequences (de Graaf et al., 2012b). But NIBS can certainly complement this.

## NIBS AND CONSCIOUS VISION

Since brain stimulation can make a unique contribution to the disentangling of empirical NCCs, it may be useful to gain an overview. In this section we therefore provide an exemplary, though non-exhaustive, review of current literature on how NIBS has contributed to our understanding of brain events underlying conscious vision. It should become clear that most if not all of this work goes beyond the concept of empirical NCCs, revealing functional relevance of brain regions both before and after visual stimulus onset and interactions between regions that influence the quality or quantity of conscious vision.

### PHOSPHENES

The simplest TMS protocol immediately reveals the value of TMS for NCC research. A single TMS pulse, applied over occipital cortex, can actually induce a conscious visual percept, called a *phosphene* (e.g., Marg and Rudiak, 1994; Kammer, 1999). This in itself is interesting, because phosphenes likely involve activity of early visual cortex, such as regions V1/V2/V3 (Thielscher et al., 2010). Unless substantial feedback from these areas to subcortical regions turns out to be paramount to conscious vision, this suggests that many subcortical processing steps elicited by exogenous visual stimulation may not be necessary for conscious experience *per se*. A simultaneous TMS-fMRI study could address the matter of subcortical responses to a perceived phosphene, but this appears to remain an empirical question.

With EEG, however, a recent study did manage to implement a strong ON–OFF paradigm to isolate phosphene-specific responses across the (surface of the) brain. Taylor et al. (2010) determined the TMS intensity that led to phosphene perception in approximately half of all trials (the phosphene threshold). By concurrently measuring EEG responses they could show that, in phosphene-present trials versus phosphene-absent trials *ceteris paribus*, widespread electrophysiological responses ranging from occipital-posterior to frontal regions were specific to conscious perception of phosphenes. These ON-specific responses became apparent quite late, starting from 160 ms after the TMS pulse, suggesting involvement of recurrent processing in conscious vision.

The necessity of recurrent processing for conscious perception of phosphenes had been shown earlier, in a landmark TMS paper by Pascual-Leone and Walsh (2001). TMS applied over the human motion area hMT/V5 is known to elicit moving phosphenes. But when single TMS pulses below phosphene threshold were administered to early visual cortex (V1/V2), thus a cortical region *earlier* in the visual hierarchy (Felleman and Van Essen, 1991), the perception of moving phosphenes from hMT/V5 pulses was diminished or abolished. Importantly, this was only the case for TMS pulses applied to early visual cortex *after* the TMS pulses to hMT/V5. A TMS pulse to V1/V2 preceding the phosphene-eliciting pulse to hMT/V5 had no effect. Thus, recurrent projections from this extrastriate area to V1/V2 were *necessary* for the conscious perception of moving phosphenes. Feedback to early visual cortex activity thus seems to be a neural prerequisite or substrate, not a consequence. In an interesting follow-up study, Silvanto et al. (2005a) reversed the paradigm, administering supra threshold TMS pulses to early visual cortex, thus inducing stationary phosphenes, and evaluating the effect of preceding sub threshold TMS pulses to hMT/V5. They could show that such sub threshold TMS pulses, too weak to elicit moving phosphenes in isolation, could nevertheless affect the quality of phosphenes elicited by subsequent supra threshold early visual cortex TMS. In short: the stationary phosphenes started moving!

Another line of research on phosphenes has involved the study of frontoparietal influences on early visual cortex. Starting with frontal cortex, the bilateral cortical regions known as the frontal eye fields (FEF) have been known to be involved in eye movements and attention (Corbetta, 1998; Corbetta and Shulman, 2002). These regions were also related to successful perception of visual stimuli, for instance in physiological studies with monkeys (Thompson and Schall, 1999). Electrical stimulation of monkey FEF neurons 50–175 ms prior to a visual stimulus actually improved detection of that stimulus (Moore and Fallah, 2001), and follow-up work demonstrated an increase in sensitivity of area V4 neurons by FEF stimulation (Moore and Armstrong, 2003). This and other work (e.g., Ekstrom et al., 2009) suggests a top-down influence of FEF on early visual regions, with the possible consequence of improved conscious vision. Using TMS over FEF in human subjects, Grosbras and Paus (2003) confirmed this hypothesis, showing that single TMS pulses over FEF preceding visual stimuli could improve visual target detection in a masking paradigm. Later work by Silvanto et al. (2006) demonstrated that TMS over FEF also directly affected human motion area hMT/V5, as TMS over FEF decreased the threshold for perception of moving phosphenes. In a similar vein, TMS applied to parietal cortex (posterior parietal cortex, PPC) decreased the threshold for stationary phosphenes elicited by TMS over early visual cortex. But, interestingly, only if PPC was stimulated in one hemisphere; bilateral PPC stimulation canceled out this effect (Silvanto et al., 2009). Below, we will return to TMS applied to frontal and parietal cortices in the study of conscious vision, but first we will address a rapidly growing field known as “TMS masking.”

### TMS OVER VISUAL CORTICES

Transcranial magnetic stimulation pulses applied to early visual cortex at rest can elicit phosphenes. But if pulses are applied to the same cortical structures (Kastner et al., 1998; Kammer, 1999), they can also disrupt ongoing processing of a visual stimulus (Amassian et al., 1989, 1993; Beckers and Hömberg, 1991; Ro et al., 2003; de Graaf et al., 2011a,c, 2012a; Koivisto et al., 2011a; Jacobs et al., 2012a,b; Salminen-Vaparanta et al., 2012) and thus abolish it from conscious perception altogether. This demonstrates that early visual cortex activations in the NCC are not consequences, but actually crucial for a conscious percept to arise. It does not, unfortunately, distinguish between substrates and prerequisites. But it does strongly inform (e.g., recurrent) models of visual awareness, due to the chronometric potential of TMS studies (for a recent review see de Graaf et al., 2014).

By applying TMS pulses at a range of stimulus onset asynchronies (SOAs), researchers could demonstrate that recurrent interactions between early visual cortex and higher-order regions are necessary for conscious perception not only of phosphenes, but also when regular visual stimuli were used (e.g., Ro et al., 2003). For example, Silvanto et al. (2005b) applied TMS pulses at different SOAs to early visual cortex or to hMT/V5, measuring conscious perception of motion stimuli. Chronometrically, first only early visual cortex TMS disrupted conscious perception of motion, then only TMS over hMT/V5 disrupted conscious perception, and subsequently only early visual cortex TMS again disrupted conscious perception of motion (see also Koivisto et al., 2010). Also for stationary stimuli, the necessity of recurrent projections from extrastriate cortex (this time lateral-occipital cortex) to early visual cortex was recently demonstrated (Koivisto et al., 2011b). A very interesting recent demonstration of how recurrent processing leads to conscious vision involved the TMS-masking of Kanizsa-type illusory stimuli. TMS pulses applied to early visual cortex *only* successfully masked such illusory percepts in SOAs *following* the SOAs in which extrastriate cortex was functionally relevant (Wokke et al., 2013).

Another interesting application of TMS in the masking paradigm involves what has been referred to as “TMS-induced blindsight” (Ro, 2010). TMS applied to early visual cortex can abolish vision, but this can be measured and evaluated in two ways: with direct subjective reports [“did you see the stimulus (feature)?”], or with (forced-choice) stimulus discrimination tasks. In contrast to blindsight patients, participants with fully intact brains have not had years of training and possible brain reorganization, so it would be useful to probe “blindsight” behavior in them. TMS has been used to this effect, and successfully demonstrated blindsight-like behavioral patterns across a range of stimuli and tasks. In trials without reported awareness of TMS-masked stimuli, “unseen” stimuli could still affect saccade responses (Ro et al., 2004), reaching movements (Christensen et al., 2008; Ro, 2008), emotion recognition (Jolij and Lamme, 2005), and even orientation and color discrimination (Boyer et al., 2005). The occurrence of such dissociations seems to depend on SOA (Koivisto et al., 2010; Jacobs et al., 2012b; Allen et al., 2014). Depending on stimuli and tasks, some have reported that TMS does affect subjective and objective measures, as well as priming measures, as a whole across SOAs (Sack et al., 2009; Jacobs et al., 2012a). And it has been shown recently that the experimental paradigm and analysis can make quite a difference as well. Lloyd et al. (2013) showed that, in their experiments, obtained TMS-induced blindsight effects disappeared when performing signal detection theory analysis, suggesting that response criteria may play a large role and should be controlled for. New evidence on this exciting topic is continually added, such as a very clever study by Allen et al. (2014) who attempted to determine whether retinotectal and/or geniculate subcortical pathways underlie TMS-induced blindsight effects, by manipulating their stimuli such that the retinotectal (and magnocellular) pathways were bypassed. They found that blindsight-like performance still obtained for such stimuli.

### TMS OVER PARIETAL CORTEX

Interestingly, we can make a smooth transition from TMS masking to TMS studies of the role of parietal cortex in conscious vision, because recent reports suggest that phosphenes can be elicited by TMS over parietal cortex as well (Marzi et al., 2009). And while one recent study did not obtain consistent visual suppression by TMS pulses over these parietal regions (Tapia et al., 2014), Koivisto et al. (2014) did report evidence for parietal (inferior parietal sulcus) TMS masking of specifically subjective conscious vision.

Quite a number of TMS studies have addressed the functional role of parietal cortex in conscious vision, even if the distinction between attention and consciousness was not always clear. In bistable vision paradigms, oﬄine TMS-induced “virtual lesions” of parietal cortex affected the rate of perceptual switching (Carmel et al., 2010; Kanai et al., 2010, 2011), where the direction of effect (increased or decreased switch rates) was found to depend on the exact parietal region stimulated (Kanai et al., 2011). Effects of online parietal TMS on binocular rivalry have also been shown (Miller et al., 2000; Zaretskaya et al., 2010). Mechanisms in parietal cortex may thus play a role in the maintenance of conscious percepts, as also suggested by the finding that parietal TMS pulses can actually induce fading of a continuous peripheral stimulus from consciousness (Kanai et al., 2008). A more general role of resource allocation among competing percepts is suggested by the finding that decreasing excitability of right parietal cortex by continuous TBS (an inhibitory rTMS protocol) rather increased the durations of target disappearance in a motion-induced blindness paradigm (Nuruki et al., 2013). Parietal cortex has further been shown functionally relevant for visual awareness in TMS studies on change blindness (Beck et al., 2006; Tseng et al., 2010), attentional blink (Kihara et al., 2007), and conscious perception of an illusory gestalt (Zaretskaya et al., 2013).

One line of TMS research on the role of parietal cortex in visual awareness we find particularly intriguing, and it concerns TMS-induced extinction (Pascual-Leone et al., 1994). Patients with “neglect,” and the symptom of “extinction,” were described above. Using TMS over parietal cortex, extinction-like behavior could be replicated in healthy participants (Pascual-Leone et al., 1994), which may be attributable to a biasing of interhemispheric competition for attentional resource allocation. Parietal TMS effects “suppressing” contralateral space and occasionally “enhancing” ipsilateral space have been found repeatedly (Seyal et al., 1995; Hilgetag et al., 2001; Meister et al., 2006; Muggleton et al., 2006; Oliveri and Caltagirone, 2006; Eshel et al., 2010; Bien et al., 2012; Szczepanski and Kastner, 2013; for a recent review on attentional enhancements by NIBS see Duecker et al., 2014). Interestingly, and in keeping with the idea of interhemispheric competition, TMS delivered over both parietal cortices simultaneously actually abolishes this effect (Dambeck et al., 2006).

### TMS OVER FRONTAL CORTEX

Parietal and frontal cortex are frequently mentioned together, as a “frontoparietal network,” because they simply do often co-occur in neuroimaging studies in general, and NCC studies particularly. For some NCC paradigms, frontal regions have subsequently been shown to be functionally relevant. For example, we already saw how TMS pulses over FEF can improve visual target detection (Grosbras and Paus, 2003). In subsequent work, TMS applied over FEF concurrently with fMRI was shown to have BOLD effects in low-level visual cortices, compatible with observed effects on psychophysics (Ruff et al., 2006). We already saw that FEF pulses facilitated perception of moving phosphenes from hMT/V5 TMS (Silvanto et al., 2006), and Amassian et al. (2008) reported frontal TMS-induced facilitation of complex phosphenes elicited by early visual cortex pulses, as well as facilitated reporting of weakly illuminated letter stimuli.

For bistable vision paradigms, there is surprisingly little published research with frontal brain stimulation. Neuroimaging has often implicated both frontal and parietal activity in perceptual switching in bistable paradigms (see references above). For example, it was shown with fMRI that specifically frontal activity occurs earlier for spontaneous percept reversals than externally induced (“replay”) reversals (Sterzer and Kleinschmidt, 2007), suggesting a causal role (Sterzer et al., 2009). On the other hand, there is recent evidence that if the gradual transition of percept reversals (Knapen et al., 2011) or percept reporting (Frässle et al., 2014) are controlled for, frontal activations conventionally obtained in bistable vision studies with fMRI may be reduced or no longer found. When it comes to NIBS, while parietal disruption leads to effects (see above), we are not aware of studies demonstrating functional relevance of frontal regions for spontaneous perceptual reversals in passive bistable vision so far. (Virtual) lesions of frontal cortex appeared to affect voluntarily induced reversal rates, though no such effect on passive viewing reversals was found (Windmann et al., 2006; de Graaf et al., 2011b). Yet the frontal lobe is large and null results remain fundamentally limited (de Graaf and Sack, 2011). It could be worthwhile to combine fMRI with neuronavigated brain stimulation in future studies to further elucidate the role of frontal regions in passive bistable vision. For the moment we dare not say whether, specifically in the context of bistable paradigms, frontal NCCs are neural consequences, prerequisites or substrates.

There does seem to be evidence for frontal involvement in visual awareness as a metacognitive process. TMS over dorsolateral prefrontal cortex (DLPFC) decreased detection of visual change (Turatto et al., 2004). FMRI research controlling for objective visual task performance found a specifically frontal activation corresponding to subjective visual report (Lau and Passingham, 2006), and in a group of frontal lesion patients, predominantly subjective visual awareness in a masking paradigm was decreased (Del Cul et al., 2009). In line with this, a theta-burst TMS study in healthy volunteers inhibited bilateral DLPFC and investigated metacognitive sensitivity to visual stimuli. Concretely, a response-bias free measure was calculated, quantifying how well participants’ subjective reports of visibility could discriminate their correct or incorrect responses to the same visual stimuli. Frontal TMS decreased this metacognitive sensitivity, and further analysis suggested that this was specifically due to decreased visibility on trials with correctly identified visual stimuli (Rounis et al., 2010).

## ADVANCED NIBS PROTOCOLS AND THE STUDY OF CONSCIOUS VISION

We have seen examples of inspiring TMS experiments that directly tested, and demonstrated, functional relevance of brain regions for conscious vision, as well as functional relevance of particular projections between brain regions for conscious vision. Evidence for the latter, while revealing, was generally indirect; for example with the inference of feedforward and feedback projections between early visual cortex and motion area hMT/V5 from the relative temporal patterns of TMS-induced perceptual disruptions (Silvanto et al., 2005b). But new paradigms of NIBS may more directly address the functional relevance of brain interactions, as we discuss below. Similarly, new protocols may widen the range of NCCs that can be tested for causal involvement in conscious perception.

### ENTRAINMENT AND PHASE COHERENCE

Starting with the latter, TMS or tDCS entrainment protocols may be used to test the causal involvement of oscillatory NCCs. The power (Thut et al., 2006; van Dijk et al., 2008) and phase (Mathewson et al., 2009) of alpha oscillations in parietal–occipital cortex, for example, has been related to attention/perception, indexing the successful detection of visual targets (see also Busch et al., 2009; Mathewson et al., 2010, 2012; de Graaf et al., 2013) and see Jensen and Mazaheri (2010), Britz and Michel (2011), and Mathewson et al. (2011) for recent reviews. The relevance of these alpha oscillations had already been probed with TMS by demonstrating that their power (Romei et al., 2008) and phase (Dugué et al., 2011) at TMS pulse onset directly reflected visual cortex excitability as measured by phosphene perception. The hypothesis arose that rhythmic TMS might actually phase-lock and/or amplify such oscillations if the rhythm was of compatible frequency (Thut et al., 2011a). Thut et al. (2011b) could indeed demonstrate that alpha-frequency TMS amplifies alpha oscillations in the brain, and Romei et al. (2010) showed that such a TMS entrainment protocol had attentional/perceptual consequences. Specifically for alpha-frequency, and in a retinotopically specific location, visual performance was enhanced, directly demonstrating the causal relevance of these oscillatory patterns for conscious vision. Rhythmic TMS was recently used to confirm also the functional relevance of alpha phase for visual perception (Jaegle and Ro, 2014). Helfrich et al. (2014) recently demonstrated, using simultaneous EEG and tACS, that alpha oscillations can successfully be induced by tACS at the same frequency, moreover with perceptual consequences. The idea of TMS (or tACS) entrainment to test functional relevance of endogenous oscillations has moreover been extended to other frequencies and paradigms (Romei et al., 2011, 2012; Neuling et al., 2012).

Another recent development in the field of NIBS is the use of tACS over different regions to bring interregional oscillations either in coherence, or out of phase. This method was pioneered by Polanía et al. (2012), who applied tACS over frontal and parietal cortex at theta-frequency (and control frequencies). Importantly, either the frontal and parietal oscillatory stimulation were at the same phase (0° phase-lag), or in anti-phase (180° phase lag). Matching prior correlational evidence from EEG, specifically the in-phase frontoparietal stimulation, at the appropriate frequency, led to enhanced task performance. Anti-phase stimulation actually resulted in a performance decrement. Thus, NIBS can now also be used to study the functional relevance of previously observed oscillations, *and* the relevance of interregional coherence of oscillations, implicated in conscious vision.

A very recent example successfully demonstrates this approach. Strüber et al. (2013) took advantage of the fact that a particular bistable visual stimulus, the “motion quartet,” involves inter hemispheric communication in one perceptual interpretation (horizontal motion across the vertical meridian) but not in the other perceptual interpretation (vertical motion across the horizontal meridian). Perceptual switches in this paradigm had previously been linked to changes in the synchronization of gamma-band oscillations, so the authors applied 40 Hz (and 6 Hz as a control) tACS to bilateral occipital cortices, at either 0 degrees phase-lag or 180° phase-lag. Specifically for anti-phasic tACS at specifically 40 Hz, they observed both perceptual effects in the form of less perceived horizontal motion and effects on coherence in the gamma-band as measured by EEG.

## CONCLUSION

A review of the literature suggests that researchers in the field of NCC are often aware of the conceptual limitations around neuroimaging-based, empirical NCCs, either implicitly or explicitly advising caution with regards to interpretations of functional roles. We believe that the explicit division of empirical NCCs into three possible functional roles, which are neural prerequisites, neural substrates, and neural consequences, remains a useful one. Review of the literature also demonstrates how valuable NIBS can be, and indeed has already been, for the enlightenment of these functional roles as a complement to neuroimaging research. The growing number of brain stimulation experiments, and the continuous development of new stimulation techniques and protocols, is a testament to the potential and value of brain stimulation. Hopefully, many more applications of NIBS in the context of NCC research can be expected. And hopefully, the framework of three NCCs will prove useful in this endeavor.

## Conflict of Interest Statement

The authors declare that the research was conducted in the absence of any commercial or financial relationships that could be construed as a potential conflict of interest.
